# The effects of Vitamin D3 supplementation on Spermatogram and endocrine factors in asthenozoospermia infertile men: a randomized, triple blind, placebo-controlled clinical trial

**DOI:** 10.1186/s12958-021-00789-y

**Published:** 2021-07-05

**Authors:** Leila Maghsoumi-Norouzabad, Ahmad Zare Javid, Anahita Mansoori, Mohammadreza Dadfar, Amirarsalan Serajian

**Affiliations:** 1 Department of Nutrition, School of Allied Medical Sciences and Nutrition and Metabolic Diseases Research Center, Ahvaz Jundishapur University of Medical Sciences, Ahvaz, Iran; 2 Department of Urology, Imam Khomeini Hospital, School of Medicine, Ahvaz Jundishapur University of Medical Sciences, Ahvaz, Iran; 3 Health Education Group, Jahad Daneshgahi, Ahvaz, Khuzestan Iran

**Keywords:** Vitamin D3, Idiopathic asthenozoospermia, Semen quality, Sex hormones

## Abstract

**Objective:**

Evaluate the effects of vitamin D3 (VD3) on sperm parameters and endocrine markers in infertile men with asthenozoospermia.

**Materials and methods:**

This randomized, triple-masking, placebo-controlled clinical trial conducted on 86 asthenozoospermia infertile men with serum 25 hydroxy vitamin D3 (25(OH)VD3) **<** 30 ng/ml in the infertility clinic of Ahvaz Jahad daneshgahi, Iran**.** Patients were randomly allocated to groups A and B, who received daily 4000 IU VD3 and matching placebo respectively for 3 months. Demographic data, dietary intake, physical activity, sun exposure, anthropometric indices, serum 25(OH)VD3, luteinizing hormone (LH), follicle-stimulating hormone (FSH), total testosterone (T), estradiol (E2),, sex hormone-binding globulin (SHBG), free androgen index (FAI = T/SHBG. 100), T/LH and T/E2 ratios, prolactin (PRO), parathyroid hormone (PTH), osteocalcin (OCN), phosphorus and sperm parameters were assessed.

**Results:**

Three months VD3 supplementation with 4000 IU/day had no significant effects body weight, body mass index (BMI), waist circumference (WC), body fat (BF), serum, OCN, LH, FSH, T, E2, SHBG, PRO, T/E2 ratio, FAI, semen volume, sperm count and normal sperm morphology*.* It increases serum 25(OH)VD3, PTH and phosphorus and seminal and serum calcium, T/LH ratio and total and progressive sperm motility and decreased significantly compared to the baseline and placebo group.

**Conclusion:**

VD3 supplementation may affect sperm motility in men with asthenozoospermia and serum 25(OH)VD3 < 30 ng/ml.

**Trial registration:**

Iran Clinical Trials Registry, ID: IRCT20151128025274N4, registered on 28 March 2018, URL of trial registry record: https://www.irct.ir/trial/29983

## Introduction

Infertility is defined as inability of a non-contracepting couple in accessing pregnancy after 12 months of regular sexual intercourses. Accordingly 15% of couples are affected by infertility worldwide [1]. As well, about 24.9% of Iranian couples have infertility problems that is higher than the global average [2]. It was indicated that about 40 to 50% of the causes of infertility are related to male factor [3]. Asthenozoospermia, as the impaired sperm motility, is involved in 19% of infertile cases. Persistent poor motility predicts failure in fertilization. Unfortunately, no specific treatment exists for improving the quality of semen in men so far [4]. Beside available therapeutic protocols in this regard, the use of dietary antioxidants has also attracted much attention in improving male fertility during recent years. Correspondingly, this may be due to that they are less costly and their positive effects have been reported on sperm oxidative damage, and increasing sperm count and motility [5–7].

One of these important antioxidant nutrients is vitamin D3 (VD3). VD3 deficiency is the most common nutritional deficiency worldwide [8]. VD3 receptors are expressed in most tissues, indicating its further roles in addition to regulating calcium and bone homeostasis [9]. The expression of VD3 receptors and its metabolic enzymes (including CYP2R1, CYP27B1, and CYP24A1), in testicles, especially in leydig cells, in epididymis, seminal vesicles, prostate, spinal cord, and head region of sperm indicate the important roles of VD3 in fertility and reproduction among men [6]. Low quality of semen in terms of sperm number, motility, and morphology as also reported in rodents with VD3 deficiency, showing the importance of vitamin D in male reproduction [10–13]. Based on available evidence in this regard, fertile men have higher VD3 levels compared to infertile men [10, 11, 13]. Notably, the results of human observational studies on the correlation between vitamin D status and sperm parameters are highly controversial. However, these studies agreed on the possibility that VD3 might have a positive effect on human male fertility potential. Moreover, some studies reported a positive association among serum VD3 levels, sperm total, progressive motility [14–17] sperm count, and normal morphology [17–21]. In contrast, some other studies reported that serum VD3 is not related to sperm count [14, 21–25] and sperm normal morphology [14, 18, 21, 22, 25–28]. In a recent cross-sectional study, Ciccone et al. showed that serum VD3 concentration is positively correlated with all sperm parameters, especially with total number of spermatozoa and progressive motility [29]. Moreover, an association between VD3 and serum androgen has been reported [29, 30]. However, the link between VD3 deficiency and serum androgen levels is poorly understood yet. In this regard, higher levels of prolactin as well as low levels of VD3 have been observed in infertile men [31, 32]. So, it has been suggested that measurements of 25 hydroxy VD3 levels and active metabolite form of VD3 (1.25 (OH) _2_ D3) should be performed, which can help in investigating the quality of sperm [9, 11]. However, the exact role of VD3 in the male reproductive biology is not fully understood yet [14].

Therefore, according to available evidence on the expression of VD3 receptors andenzymes involved in the metabolism of VD3 in the genital and male sperm, whichcan reflect the roles of this vitamin in spermatogenesis, sperm maturation, endocrinefunctions, and thereby in the improvement of male fertility, and also according to the high prevalence of VD3 deficiency in Iran, we hypothesize that VD3 supplementation in men with idiopathic asthenozoospermia and insufficient vitamin D3 levels or VD3 deficiency, may affect their sperm parameters especially sperm total and progressive motility and also endocrine factors that are involved in male fertility. Accordingly, progressive motility as one of the most important indicators of sperm quality provided by basic semen analysis, has a positive predictive value for natural conception and even intrauterine insemination (IUI) [33]. It was reported that positive effects are observed on these parameters, so VD3 as a simple, less invasive, and cost effective clinical measurement, could be used in future infertility treatment and assisted reproductive technologies (ART). Therefore, further investigations, including randomized, controlled, clinical trials as well as in vitro and in vivo studies, are needed to explore the therapeutic potential of VD 3 supplementation fully in cases of male infertility. Up to now, very few clinical trials have examined the effects of VD3 supplementation on male infertility, and reported controversial results. Furthermore, most of these studies have used VD3 and calcium supplementations together, not only VD3. So the main objective of the present study was to evaluate the effects of VD3 supplementation on sperm parameters and serum endocrine factors in men with idiopathic asthenozoospermia.

## Materials and methods

### Participants

The present randomized, triple-masking, placebo-controlled clinical trial was conducted on 86 asthenozoospermia infertile men (the mobility of sperm < 40% and rapid progressive sperm motility < 32% [34])with VD3 levels less than 30 ng / ml in the infertility clinic of Ahvaz Jahad daneshgahi, Iran, from October 2018 to August 2020. The patients who met the specific inclusion criteria, comprising inability to have a child after at least 1 year of marriage without the use of any preventive measure, normal fertile woman partner, no medical history that could cause infertility, no drug or alcohol abuse, no job and environmental exposure to toxins that can cause infertility [35], a body mass index (BMI) less than 30 kg/m^2,^ and no medical therapy ≤3 months prior to research initiation, were recruited in this research. Moreover, the exclusion criteria included any acute illness during the study, admission of less than 90% of the supplement, leaving the study based on the personal desire of the participants, attendance at another research study, immigration, and unavailability for follow-up. Additionally, those men with varicocele [36], epididymo-orchitis [37], sexually transmitted diseases [38], and systemic diseases [39] were excluded based on the proved negative effects of the above-mentioned pathologies on male fertility and sperm motility [40]. Furthermore, men with endocrinopathies such as hyperthyroidism and hypothyroidism were excluded as well, because hyperthyroidism is associated with the reduced semen volume and reduced sperm density, motility, and morphology, and hypothyroidism is associated with the reduced sperm morphology [41, 42].

### Randomization and blinding and interventions

**Fig. 1:**
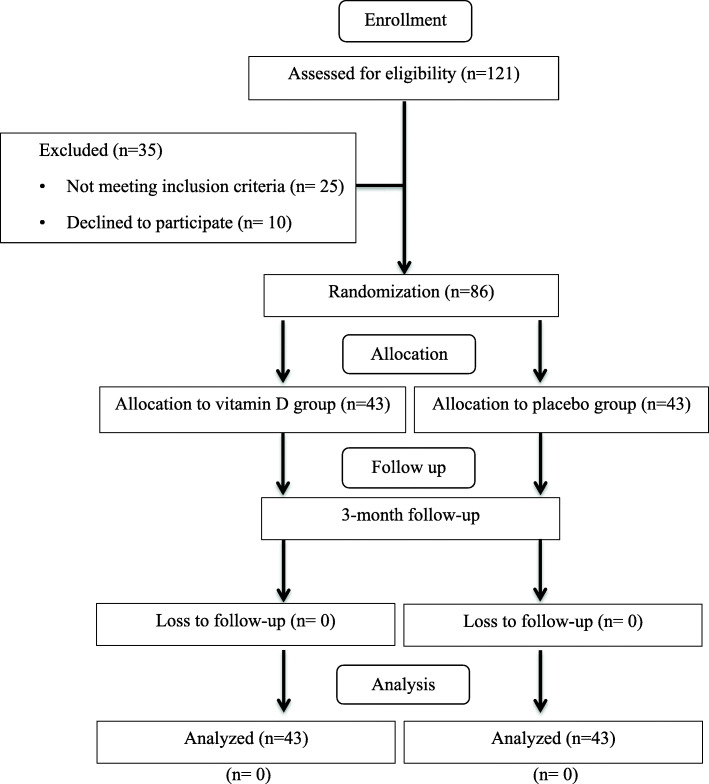
Study flow diagram

Before starting the study, the patients were randomly allocated to the following groups: group A, who received daily doses of 4000 IU [43, 44] VD3 (cholecalciferol); and group B, who received identical placebo for 3 months (Figs. 1 and 2). Afterward, each participant received a randomization number, which was calculated using a computer-generated program. Next, a blocked randomization table was generated using random permuted blocks design with a block size of six. A third party who was not from the study investigators team performed the research randomization. To decrease the probability of bias, the investigator, statistical analyzer, and patients were masked to the treatment situation. The VD3 and placebo containing maltodextrin were supplied by Ahvaz Jundishapur University of Medical Sciences Pharmaceutical Technology Development Center, Iran. Of note, to sustain and guarantee masking, VD3 and placebo were matched in appearance. At the end of the study, when all the patients had completed the study, the codes related to each group were opened.

### Measurements

The patients were interviewed and a demographic questionnaire was filled out at the beginning of the study. Dietary intake was then assessed using three-day 24-h record questionnaires, including two week days and one weekend day once at the beginning and once at the end of the study. Thereafter, total intakes of energy, macronutrient, and micronutrient were estimated using nutritionist IV software. Physical activity levels were also evaluated using the short form of the international physical activity questionnaire (IPAQ) [45] at the beginning and at the end of the study. The length of sun exposure, the time of the day spent by the patients outside, exposed parts of the body to sunlight, and sunscreen use were collected at baseline and at the end of the study [46]. Anthropometric indices were also evaluated after overnight fasting, with minimal clothing and no shoes, once at baseline and once at the end of the study. As well, the patients’ body weight was measured in minimum clothing and without shoes using Omron scale (HBF-500, Germany) with an accuracy of 0.1-kg. Afterward, the patients’ body fat percent was measured using Omron scale (HBF-500, Germany). The Omron full body sensor body composition monitor and scale was used to estimate the body fat percentage by the bioelectrical impedance method and by considering the patients’ height, weight, age, and gender. The patients’ height was measured without shoes using a stadiometer with an accuracy of 0.5- cm and BMI was calculated by dividing the weight in kilograms (kg) by the height squared in meters (m^2^). Biochemical parameters consist of serum and semen samples that were taken both at the beginning and after the intervention**.**

### Semen samples preparation

Before and after intervention patients’ two semen samples separated by a median of 10 days, were collected after three days of sexual abstinence and kept at 37 °C to become liquid. The samples were then analyzed according to the criteria of the World Health Organization (WHO) [34] and sperm quality parameters including semen volume, total sperm count, sperm motility and sperm with normal morphology (%) were assessed. The rest of the liquefied semen samples were immediately centrifuged at 300 rpm for 10 min to separate the seminal plasma. The seminal plasma samples were then divided into several aliquot parts and stored in − 80 °C for further biochemical analysis such as calcium.

Furthermore at before and after intervention, 10 cc of intravenous blood samples were taken from patients and centrifuged and kept at − 80 °C until biochemical analysis including 25(OH)VD3, Sex hormones (total testosterone, estradiol (E2), luteinizing hormone (LH), follicle-stimulating hormone (FSH), sex hormone-binding globulin (SHBG), prolactin (PRO), parathyroid hormone (PTH), osteocalcin (OCN), calcium, and phosphorus.

Measurement of serum calcium and phosphorus and seminal fluid calcium were done using calorimetric method. Serum VD3 levels were assessed by enzyme-linked immunosorbent assay (ELISA and Monobind kit (Monobind, USA). PTH and osteocalcin were measured by ELISA and Biomerica kit (Biomerica, USA). T, E2 and SHBG were measured by ELISA and Diametra kit (Diametra, Italy). Luteinizing hormone (LH), follicle-stimulating hormone (FSH), and prolactin were measured by ELISA and Pishtaz Teb kit (Pishtaz Teb, Iran).

### Analysis

Data were analyzed by SPSS (Version 22; SPSS Inc., Chicago, IL). Mean ± SD of data were reported for quantitative and frequency and (percentage) were reported for qualitative variables. Kolmogorov-Smirnov test was used for assessing the normal distribution of variables. Independent sample t-test or Mann-Whitney U test were used to analyze the baseline differences of mean values of variables with normal or non- normal distribution respectively. Analysis of covariance (ANCOVA) was used to determine any between group differences between post intervention, adjusting for baseline values and covariates (including BMI, age, physical activity levels, sunlight exposure, season, dietary intake of macro and micronutrients, serum VD3, PTH and calcium). Paired sample t tests and Wilcoxon signed-rank test “as nonparametric alternatives” were used to evaluate within groups comparison of mean values for normal and non- normal distribution variables respectively, pre- and post-intervention. *P* < 0.05 was considered statistically significant.

## Results

### Basic characteristics of the research participants

In this research, 86 patients were recruited and completed the research (12 weeks) without any side effects: 43 in the VD3 group and 43 in the placebo group (Fig. 1). Table shows basic characteristics of the research subjects. No significant differences in marriage duration, education, race, physical activity, sun exposure, and sun exposure parts of the body of participants and sun screen use were seen between the two groups (*P* > 0.05). The mean age of subjects (35.13 ± 5.51 vs. 34.44 ± 5.07 years) and mean infertility duration (2.75 ± 2.25 vs. 3.27 ± 1.97) did not differ between two groups (*P* > 0.05). Also, weight, BMI, WC and BF percentage means did not vary significantly between two groups at baseline and end of the study (*P* > 0.05) (Table 1).

**Table 1:** Characteristics of the trial participants

Variables	Time		Vitamin D3 (*n* = 43)	Placebo (*n* = 43)	*P* value (between)
Age (y)			35.13 ± 5.51	34.44 ± 5.07	0.566^a^
Marriage Duration (y)			4.65 ± 2.26	5.32 ± 3.45	0.288^a^
Infertility Duration (y)			2.75 ± 2.25	3.27 ± 1.97	0.256^a^
Education	Elementary		1(2.3)	1 (2.3)	0.506^b^
	Diploma		11 (25.6)	13 (30.2)	
	Associate Degree		10 (23.3)	14 (32.6)	
	Bachelor		6 [14]	5 (11.6)	
	Master’s degree		11 (25.6)	5 (11.6)	
	Ph.D.		4 (9.3)	5 (11.6)	
Race	Fars		7 (16.3)	13 (30.2)	0.215^b^
	Arab		22 (51.2)	13 (30.2)	
	Lor		12 (27.9)	14 (32.6)	
	Tork		2 (4.7)	3 (7)	
Height (cm)			173.48 ± 6.05	172.79 ± 7.66	0.641^a^
Weight (kg)	Baseline		85.80 ± 12.00	83.63 ± 10.48	0.374^a^
	12 weeks		85.67 ± 11.85	83.33 ± 10.95	0.345^a^
	Change		-0.12 ± 0.66	− 0.29 ± 2.06	0.615^a^
*P* value (within)^c^			0.209	0.353	
BMI (kg/m^2^)	Baseline		28.40 ± 2.96	27.95 ± 2.51	0.452^a^
	12 weeks		28.36 ± 2.91	27.84 ± 2.63	0.387^a^
	Change		−0.03 ± 0.22	− 0.11 ± 0.72	0.525^a^
*P* value (within)^c^			0.272	0.313	
WC (cm)	Baseline		105.58 ± 8.45	104.32 ± 8.21	0.487^a^
	12 weeks		105.34 ± 8.06	104.23 ± 8.88	0.656^a^
	Change		−0.53 ± 1.16	− 0.09 ± 1.87	0.193^a^
*P* value (within)^c^			0.133	0.747	
BF percentage	Baseline		31.23 ± 8.77	31.13 ± 6.47	0.950^a^
	12 weeks		31.09 ± 8.68	30.97 ± 6.21	0.943^a^
	Change		−0.14 ± 0.48	− 0.15 ± 0.54	0.913^a^
*P* value (within)^c^			0.053	0.064	
Physical Activity	Baseline	Low	18 (41.9)	20 (46.5)	0.664^b^
		average	25 (58.1)	23 (53.5)	
	12 weeks	Low	21 (48.8)	22 (51.2)	0.829^b^
		average	22 (51.2)	21 (48.8)	
*P* value (within)^d^			0.581	0.727	
Sun exposure	Baseline	< 1 h	13 (30.23)	12 (27.90)	0.812^b^
		> 1 h	30 (69.76)	31 (72.09)	
	12 weeks	< 1 h	15 (34.88)	15 (34.88)	1.00^b^
		> 1 h	28 (65.11)	28 (65.11)	
*P* value (within)^d^			0.765	0.579	
Time of sun exposure	Baseline	10 AM–3 PM	23 (53.48)	30 (69.76)	0.326^b^
		Other	20 (46.51)	14 (32.55)	
	12 weeks	10 AM–3 PM	21 (48.83)	27 (62.79)	0.553^b^
		Other	22 (51.16)	16 (37.20)	
*P* value (within)^d^			0.683	0.447	
Sun exposure area	Baseline	Face and Hand	14 (32.6)	16 (37.2)	0.651^b^
		Face and Hand and Arm	29 (67.4)	27(62.8)	
	12 weeks	Face and Hand	19 (44.18)	21 (48.83)	0.420^b^
		Face and Hand and Arm	24 (55.81)	22 (51.16)	
*P* value (within)^d^			0.228	0.138	
Sun screen use	Baseline	Yes	17 (39.53)	16 (37.20)	0.612^b^
		No	26 (60.46)	27 (62.79)	
	12 weeks	Yes	18 (41.86)	19 (44.18)	0.586^b^
		No	25 (58.13)	24 (55.81)	
*P* value (within)^d^			0.958	0.321	

BMI, body mass index; WC, waist circumference; BF, body fat

- Values are expressed as mean ± standard deviation (SD) or Frequency and percent

- Between group differences pre intervention, the *p* value are reported based on the independent sample *t* test (a) or Chi-Square test (b)

- Between group differences post intervention; the *p* value are reported based on Chi-Square (b)

- Between groups comparing the changes of variables, the *p* value is reported using the independent sample *t* test (a)

- Within group differences, the *p* value is reported using the paired *t* test (c) or McNemar (d)

-*P* < 0.05 was considered significant

### Serum 25(OH)VD3, PTH and OCN concentration

Table 2 shows the mean ± SD of the serum 25(OH)VD3, PTH and OTC before and after intervention in both groups. There was no significant difference in the baseline mean of serum 25(OH)VD3, OCN and PTH between two groups (*p* > 0.05). After 3 months of the intervention the mean changes of 25(OH)VD3 increased and the mean changes of PTH decreased significantly in VD3 group compared with placebo group (14.00 ± 2.17 vs. -0.23 ± 2.15; *P* < 0.001, 1 and − 2.37 ± 1.36 vs. 0.41 ± 1.54; *P* < 0.001 respectively). But the mean of OCN did not changed significantly at the end of the study (*p* > 0.05). ANCOVA model adjusted for baseline values, BMI, physical activity and energy, did not change the *p*-value significance. Also, paired t-test results demonstrated a significant increase in serum 25(OH)VD3 and a significant decrease in PTH in VD3 group compared with baseline (32.3 ± 6.39 vs. 18.30 ± 6.87; *P* < 0.001, and 29.96 ± 10.74 vs. 32.33 ± 11.17; *P* < 0.001 respectively). But there was no significant within group changes in OCN levels in both groups (*p* > 0.05).

**Table 2:** Between group comparison of serum concentration of 25(OH)VD3, calcium, phosphorous and hormones and seminal calcium pre and post intervention

Variables	Time	Vitamin D3 (*n* = 43)	Placebo (*n* = 43)	*P* value (between)
25(OH)VD3 (ng/ml)	Baseline	18.30 ± 6.87	19.17 ± 6.42	0.545^a^
	12 weeks	32.30 ± 6.39	18.93 ± 7.00	< 0.001^b^
	Change	14.00 ± 2.17	− 0.23 ± 2.15	< 0.001^c^
*P* value (within)^d^		< 0.001	0.482	
PTH (pg/ml)	Baseline	32.33 ± 11.17	31.34 ± 13.67	0.714^a^
	12 weeks	29.96 ± 10.74	31.76 ± 13.22	< 0.001^b^
	Change	−2.37 ± 1.36	0.41 ± 1.54	< 0.001^c^
*P* value (within)^d^		< 0.001	0.082	
OCN (ng/ml)	Baseline	11.82 ± 4.52	12.42 ± 4.16	0.525^a^
	12 weeks	11.94 ± 4.61	12.35 ± 4.12	0.663^b^
	Change	0.11 ± 0.57	−0.06 ± 0.45	0.100^c^
*P* value (within)^d^		0.185	0.334	
Tt (ng/ml)	Baseline	3.87 ± 2.08	3.93 ± 2.04	0.884^a^
	12 weeks	3.95 ± 1.75	3.86 ± 2.01	0.482^b^
	Change	0.07 ± 0.64	− 0.07 ± 0.36	0.177^c^
*P* value (within)^d^		0.416	0.199	
SHBG (nmol/l)	Baseline	18.80 ± 3.60	19.16 ± 4.68	0.692^a^
	12 weeks	19.09 ± 3.69	18.88 ± 4.71	0.815^b^
	Change	0.29 ± 1.22	−0.27 ± 1.55	0.062^c^
*P* value (within)^d^		0.125	0.245	
E2 (pg/ml)	Baseline	26.70 ± 12.46	25.67 ± 10.87	0.684^a^
	12 weeks	26.54 ± 12.53	25.61 ± 10.86	0.095^b^
	Change	−0.15 ± 0.54	−0.05 ± 0.32	0.311^c^
*P* value (within)^d^		0.065	0.254	
PRO (mIU/L)	Baseline	300.13 ± 195.55	279.50 ± 158.56	0.592^a^
	12 weeks	294.57 ± 188.97	280.15 ± 158.14	0.265^b^
	Change	−5.56 ± 21.22	0.64 ± 18.25	0.149^c^
*P* value (within)^d^		0.093	0.818	
LH (IU/L)	Baseline	5.41 ± 4.13	5.06 ± 2.21	0.627^a^
	12 weeks	5.40 ± 4.09	5.10 ± 2.25	0.186^b^
	Change	−0.01 ± 0.26	0.03 ± 0.18	0.324^c^
*P* value (within)^d^		0.646	0.292	
FSH (IU/L)	Baseline	4.80 ± 4.90	4.05 ± 3.45	0.417^a^
	12 weeks	4.78 ± 4.87	4.06 ± 4.43	0.116^b^
	Change	−0.02 ± 0.15	0.01 ± 0.11	0.089^c^
*P* value (within)^d^		0.076	0.123	
FAI (T/SHBG. 100) (%)	Baseline	42.54 ± 35.27	39.61 ± 23.13	0.650^a^
	12 weeks	40.56 ± 22.71	39.13 ± 23.72	0.207^b^
	Change	−1.97 ± 18.59	−0.48 ± 6.14	0.619^c^
*P* value (within)^d^		0.490	0.608	
T/LH ratio	Baseline	1.22 ± 1.48	0.88 ± 0.53	0.159^a^
	12 weeks	1.32 ± 1.58	0.88 ± 0.56	0.142^b^
	Change	0.09 ± 0.29	−0.003 ± 0.11	0.037^c^
*P* value (within)^d^		0.032	0.585	
T/E2 ratio	Baseline	0.16 ± 0.10	0.17 ± 0.08	0.882^a^
	12 weeks	0.17 ± 0.08	0.16 ± 0.07	0.360^b^
	Change	0.005 ± 0.03	−0.002 ± 0.01	0.203^c^
*P* value (within)^d^		0.316	0.419	
Serum Ca (mg/dl)	Baseline	9.17 ± 0.43	9.28 ± 0.69	0.408^a^
	12 weeks	9.21 ± 0.43	9.26 ± 0.70	0. 151^b^
	Change	0.03 ± 0.08	−0.02 ± 0.11	0.009^c^
*P* value (within)^d^		0.008	0.211	
Semen Ca (mg/dl)	Baseline	12.27 ± 3.64	12.72 ± 4.14	0.594^a^
	12 weeks	12.47 ± 3.60	12.57 ± 4.13	0.218^b^
	Change	0.15 ± 0.46	−0.12 ± 0.72	0.038^c^
*P* value (within)^d^		0.035	0.270	
*P* (mg/dl)	Baseline	3.52 ± 0.72	3.73 ± 0.79	0.219^a^
	12 weeks	3.62 ± 0.71	3.65 ± 0.8	0.470^b^
	Change	0.04 ± 0.11	−0.08 ± 0.41	0.049^c^
*P* value (within)^d^		0.009	0.190	

25(OH)VD3, 25-hydroxy vitamin D3; PTH, parathyroid hormone; OCN, osteocalcin; T, testosterone; SHBG, sex hormone-binding globulin; E2, estradiol; FAI, free androgen index; PRO, prolactin; LH, luteinizing hormone; FSH, follicle-stimulating hormone; Ca, calcium and P, phosphorus

- Values are reported as mean ± standard deviation or Frequency and percent

- Between groups differences pre intervention, the *p* value are reported using the independent sample *t* test (a)

- Between groups differences post intervention; the *p* value is reported using the analysis of covariance (ANCOVA) (b)

- Between groups comparing the changes of variables, the *p* value is reported using the independent sample *t* test (c)

- Within group difference, the *p* value is reported using the paired *t* test (d)

-P < 0.05 was considered significant

### Serum and seminal calcium and serum phosphorus

Table 2 shows the mean ± SD of the serum and semen calcium before and after intervention in both groups. No significant difference in the mean of serum and seminal calcium and serum phosphorus were observed at the beginning of the study (*P* > 0.05). VD3 supplementation in intervention group increased mean change of serum and seminal calcium and serum phosphorus significantly compared with placebo group (0.03 ± 0.08 vs. -0.02 ± 0.11; *P* = 0.009, 0.15 ± 0.46 vs. − 0.12 ± 0.72; *P* = 0.038 and 0.04 ± 0.11 vs. -0.08 ± 0.41; *P* = 0.049 respectively). The results remained significant even after adjusting for the effect of confounding factors. Within-group changes also demonstrated a significant increase in levels of serum and seminal calcium and serum phosphorus in VD3 group compared with baseline (9.21 ± 0.43 vs. 9.17 ± 0.43; *P* = 0.008, 12.47 ± 3.60 vs. 12.27 ± 3.64; *P* = 0.035, and 3.62 ± 0.71 vs. 3.52 ± 0.72; P = 0.009 respectively).

### Serum T, SHBG, E2, PRO, LH and FSH, FAI, T/LH and T/E2 ratio

Independent sample t test showed no significant differences in the mean of serum T, SHBG, E2, PRO, LH, FSH, FAI, and T/E2 ratio between groups at baseline and end of the study (*P* > 0.05) (Table 2). Mean of T/LH ratio were not different between groups at baseline (*P* > 0.05), but mean change of T/LH ratio increased significantly in VD3 group in comparison to the placebo group at the end of the study (0.09 ± 0.29 vs − 0.003 ± 0.11; *P* = 0.037). The results remained significant even after adjusting for the effect of confounding factors. Within-group changes also showed no significant changes in serum T, SHBG, E2, PRO, LH, FSH levels, FAI, T/E2 and T/LH ratios in both groups in comparison to the baseline (*P* > 0.05).

### Semen parameters (volume, sperm count, total motility, progressive sperm and sperm with normal morphology)

Table 3 presents the parameters of semen of the infertile patients pre and post intervention. No significant differences were observed in mean of ejaculate volume, sperm count, total motility, progressive sperm and sperm with normal morphology between the two groups at the baseline (*P* > 0.05). However, supplementation with VD3 increased significantly mean changes of sperm total motility and progressive sperm in comparison to the placebo group (5.06 ± 5.77 vs. -1.44 ± 7.87; *P* < 0.001, and 8.84 ± 5.89 vs. -0.86 ± 3.51; *P* < 0.001, respectively). The results remained significant even after adjusting for the effect of confounding factors. Within-group analyses indicated that sperm total motility and progressive sperm increased significantly after intervention in the VD3 group (38.72 ± 8.42 vs. 33.65 ± 7.99; *P* < 0.001, and 23.41 ± 8.62 vs. 18.56 ± 6.41; *P* < 0.001). Other sperm parameters, including ejaculate volume, sperm count and sperm with normal morphology, did not change significantly between the two groups (*P* > 0.05).

**Table 3:** Between group comparison of comparison of sperm parameters pre and post intervention

Variables	Time	Vitamin D3 (*n* = 43)	Placebo (*n* = 43)	*P* value (between)
Volume (ml)	Baseline	3.75 ± 1.49	4.13 ± 1.86	0.296^a^
	12 weeks	3.84 ± 1.24	4.07 ± 1.63	0.265^b^
	Change	−0.09 ± 0.42	−0.05 ± 0.34	0.076^c^
P value (within)^d^		0.162	0.272	
Sperm count (˟ 10^6^)	Baseline	64.11 ± 10.63	65.60 ± 11.66	0.538^a^
	12 weeks	66.04 ± 10.96	65.20 ± 10.65	0.721^b^
	Change	1.93 ± 6.27	−0.39 ± 5.95	0.081^c^
P value (within)^d^		0.051	0.664	
Motile sperm (%)	Baseline	33.65 ± 7.99	34.30 ± 6.22	0.675^a^
	12 weeks	38.72 ± 8.42	32.86 ± 7.86	0.800^b^
	Change	5.06 ± 5.77	−1.44 ± 7.87	< 0.001^c^
P value (within)^d^		< 0.001	0.236	
Progressive sperm (%)	Baseline	18.56 ± 6.41	19.06 ± 4.89	0.685^a^
	12 weeks	23.41 ± 8.62	18.18 ± 5.88	0.955^b^
	Change	8.84 ± 5.89	−0.86 ± 3.51	< 0.001^c^
P value (within)^d^		< 0.001	0.142	
Normal morphology (%)	Baseline	7.53 ± 2.98	8.20 ± 2.79	0.283^a^
	12 weeks	7.79 ± 2.57	8.04 ± 2.34	0.288^b^
	Change	0.25 ± 1.27	−0.16 ± 1.02	0.097^c^
P value (within)^d^		0.195	0.302	

- Values are reported as mean ± standard deviation or Frequency and percent

- Between groups differences pre intervention, the *p* value are reported using the independent sample *t* test (a)

- Between groups differences post intervention; the *p* value is reported using the analysis of covariance (ANCOVA) (b)

- Between groups comparing the changes of variables, the *p* value is reported using the independent sample *t* test (c)

- Within group differences, the *p* value is reported using the paired *t* test (d)

-P < 0.05 was considered significant

## Discussion

The term “asthenozoospermia” refers to the individuals that spermatozoa in their semen sample being less motile than the WHO cut off value. There are some defects in the protein structure or genetic disorder causing asthenozoospermia [40], including defects in the flagella and cilia [47], defects in the sperm channels located in the sperm tail membrane like Cation channels of sperm (CaTSper channels) [48]; defects in ATP production by Glycolysis and oxidative phosphorylation (OXPHOS) in the flagellum’s central piece, head, and mitochondria [49]; and reduction in the cyclic AMP that is essential for regulating sperm motility [50]. These cellular and molecular factors may be associated with many idiopathic male infertility cases, especially idiopathic asthenozoospermia [51]. Furthermore, there are genetic mutations such as immotile cilia syndrome [52], Primary ciliary dyskinesia” (PCD) [53], and dysplasia of the fibrous sheath (DFS) [54] exerting negative effects on spermatozoa development and motility, which lead to fertility. Beyond the defects in the protein structure or genetic disorder, it was indicated that some physiological processes during sperm maturation or ejaculation, could affect sperm motility. Lifestyle changes, including dietary deficiencies, smoking, alcohol abuse, and psychological stress, exposure to chemical pesticides and air pollution, and aging can also disrupt sperm motility [55, 56]. These factors were found to be positively correlated with oxidative stress and the production of reactive oxygen species (ROS) and in turn with sperm immobility [57]. Uncontrolled apoptosis, as another physiological event, may contribute into low sperm motility [58]. Appropriate treatment is not available yet for reversing the morphological and/or motility defects observed in CaTSper-related asthenozoospermia. Additionally, no treatment is available for immotile spermatozoa because of genetic causes [59, 60]. However, protecting the quality of spermatozoa, of which sperm motility is a part, is possible. As mentioned earlier, there are some components correlated with sperm oxidative stress, which can be totally avoided. Accordingly, these include specific toxic environmental exposures; unhealthy lifestyle habits, including physical inactivity, recreational toxins, and excessive use of personal technologies, and several pathologies related to endocrine and cardiovascular diseases. In this regard, some studies have previously shown that changes in lifestyle can significantly improve both semen quality and motility [61]. Antioxidant therapy is widely used for improving sperm quality, including its motility [60, 62]. Although vitamin D is known as one of the strongest antioxidants available [63], and its deficiency is common among people worldwide, unfortunately little clinical research has been done on its effects on male fertility so far.

In the present study, 12 weeks supplementation of 4000 IU VD3 increased the mean serum concentration of 25(OH)VD3 on average 32 ng/ml in the intervention group, which can be considered as optimal [8]. By the end of the present study, the placebo group had a mean 25(OH) VD3 concentration of 18 ng/ml, which was significantly lower than that of the intervention group. The observed difference was also reflected in serum concentrations of serum calcium and PTH, showing that the intervention was efficient.

An inverse association between serum 25(OH) VD3 and PTH has been well-established. These two markers are used together as the indicators of vitamin D sufficiency [64]. Notably, no changes were found in serum 25(OH) VD3 after the supplementation with 4000 IU/day of VD3 for six months in the Lewis et al.’s study [65]. Additionally, Backx et al. in their study reported similar results within three-month supplementation of 2200 IU VD3; however, higher concentration of 25(OH) VD3 was observed after 12 months of intervention [66]. In contrast, there are studies reporting the increased serum 25(OH)VD3 concentration compared to the baseline after 12 weeks of the supplementation with 2000 IU/day of VD3 [67, 68]. It has been shown that inter-individual differences and genetic variation in VD3 binding protein affect the response to this supplementation [69].

In the present study, although VD3 supplementation had no significant effects on semen volume, sperm count, and normal sperm morphology, it significantly increased serum phosphorus and seminal and serum calcium, and total and progressive sperm motility compared to the baseline and to the placebo group.

Some correlations have been reported between VD3 deficiency and low semen quality in previous studies performed on both men and animals [10, 11, 13, 70–72]. Studies in mice model demonstrated that VDR and 1a-hydroxylase knockout mice had VD3 deficiency and reduced circulating calcium levels as well as the impairment of spermatogenesis, along with a significant decreasing in both the number and motility of sperm in comparison to the wild-type group [70, 72]. It was also shown that the normalization of serum calcium and phosphorus levels inversed the pathological changes, which affected spermatogenesis and sperm qualities [72]. These results support the hypothesis that VD3 deficiency has some adverse effects on male fertility by interfering with calcium homeostasis, and thereby reducing the amount of calcium in the reproductive system [13, 16, 72, 73]. In agreement with the result of this study, several studies have shown that VD3 serum levels are important for semen quality [15, 74]. Blomberg Jensen et al. in their study also explained that VD3 serum levels are positively associated with sperm motility, and men with VD3 deficiency had lower proportions of total and progressive motile sperms compared to men with high VD3 levels [15]. Moreover, two other studies on fertile and infertile men showed that men with vitamin D3 sufficiency had more motile spermatozoa than those with vitamin D3 deficiency [16, 17]; however, another study reported that serum vitamin D3 levels have no relationship with semen parameter values in a fertile population. While in patients with oligoasthenozoospermia or teratozoospermia, a positive correlation was found between vitamin D3 and sperm motility [14] In a recent cross-sectional study conducted by Ciccone et al., it was shown that each “one unit” (ng ml-1) increase in 25(OH)VD3 serum concentrations is correlated with a 2.1% increase in progressive motile spermatozoa in the ejaculate of 124 normozoospermic and 136 men with seminal abnormalities [29]. In the present study, each “one unit” (ng ml-1) increase in 25(OH) VD3 serum concentrations caused a 3.4% increase in progressive motile spermatozoa.

The efficacy of VD3 on human spermatozoa was also studied in several experimental studies. Blomberg Jensen et al. in their study exposed semen samples of 40 men from general population to 1, 25(OH)_2_D3 for 45 min and as a result, indicated the increased sperm motility and acrosome reaction. They reported that these effects can be mediated by increasing in intracellular calcium amount in human spermatozoa via VDR-mediated releasing of calcium from the intracellular calcium pool [23]. Additionally, another in-vitro study reported that the incubation of spermatozoa with 1,25(OH)_2_D3 for 30 min, remarkably increased motility and upward migration of spermatozoa by increasing the ATP synthesis through both the cAMP/PKA (cyclic adenosine monophosphate (cAMP) and the activation of protein kinase A (PKA)) pathways and the increased intracellular calcium ions [27]. These pathways are very important for motility of sperm, capacitation processes, and acrosome reaction, which are considered as essential factors involved in a proper spermatozoa function. In a recent in-vitro study done by Taheri Moghadam et al., the VD3 effects were investigated under in vitro condition like ART, on sperm qualities (including morphology, motility, and chromatin integrity) and apoptosis in healthy and asthenozoospermic men. As a result, they reported that VD3 has no effect on sperm normal morphology improvement; however, it could improve motility by decreasing apoptosis and sperm necrosis, especially in asthenozoospermic men. Therefore, it could be used for therapeutic opportunities [75]. Furthermore, the activation of Ca-dependent kinases and protein serine/threonine/tyrosine phosphorylation, the control of cell volume and osmolarity [60], and the improvement of integrity of sperm membrane by decreasing ROS [76], are known as some of the others proposed mechanisms for the increased sperm motility. It was found that most of the mechanisms involved in sperm motility lead to the increased intracellular Ca^2+^. Several Ca^2+^-permeable-specific channels such as high voltage-gated Ca2+ channels, cyclic nucleotide-gated channels, and transient receptor potential channels have been observed in sperm. However, the most significant sperm ion channel is the cation channel of sperm (CatSper), which is regarded as a sperm-specific Ca^2+^ channel required for the hyperactivation of sperm motility. Notably, the role of other ion channels in the spermatozoa is to ensure both the activation and modulation of CatSper [77]. The increased level of calcium may affect glycolysis and the axoneme activities, thereby promoting hyperactivation of motility. Further levels of Ca^2+^ regulate the atypical soluble adenylyl cyclase (sAC), which generates cAMP and then activates protein kinase A (PKA). PKA induces phosphorylation of axonemal dynein, leading to the consumption of ATP, which consequently increases the pHi. PKA activates sperm tyrosine kinases (with serine and threonine residues), in order to trigger a cascade of protein phosphorylation involved in sperm motility. As well, Calcium is known to be involved in the regulation of cell volume by the activation of Ca2 + −dependent K+ channels [60]. It is also involved in the regulation of seminal osmolarity. It was indicated that sperm motility is negatively affected by seminal osmolarity, as patients with normal motility exhibit a significantly lower mean value of semen osmolarity (Ca2+: 3.36 mmol l-1; osmol: 318 mmol kg-1) than that of patients with low sperm motility (Ca2+: 3.10 mmol l-1; osmol: 345 mmol kg-1) [78]. In addition, Calcium/calmodulin signaling is another proposed pathway for regulating sperm motility. Calmodulin is a key axonemal Ca2+ sensor, and the calmodulin-dependent kinase may mediate this Ca2+ signal. Calmodulin regulates motility through having a direct interaction with protein kinases, phosphatases, and sAC [60, 79]. Furthermore, calcium is also involved in the regulation of flagella movement. At low intracellular Ca2+ concentrations, flagella symmetrically beat, but when Ca2+ levels rise in the activated sperm (Ca2+ of 10–40 nM), the waveform becomes more asymmetric and sperm also becomes hyperactivated (Ca2+ of 100–300 nM) [60, 80].

Similar to the previous studies, in the present study, 12 weeks of VD3 supplementation increased sperm both total and progressive motilities as well as serum and seminal calcium and serum phosphorous compared to the baseline and to the placebo group. The result also confirms the possible hypothesis of the calcium mediated effects of VD3 on increasing sperm motility. However, further investigations, including randomized, controlled, clinical trials as well as in vitro and in vivo studies, are needed to explore the therapeutic potential of VD 3 supplementation fully in cases of male infertility.

Up to now, only three interventional studies have evaluated the effects of VD3 on sperm parameters, and reported controversial outcomes. The results of the present study are in line with the results of the Deng XL et al.’s study investigating the efficacy of a 3-month supplementation with VD3 200 IU/daily and calcium 600 mg once a day, in oligo-asthenozoospermia infertile men. As a result, they indicated a significant increase in sperm progressive motility and rate of pregnancy in the intervention group in comparison to the untreated men [24]. In contrast, Blomberg Jensen et al. [28] in their randomized clinical trial studied the effects of an initial oral dose of 300,000 IU of cholecalciferol, followed by a 150-day supplementation with 1400 IU of cholecalciferol and 500 mg of calcium or placebo in infertile men. Thereafter, no significant differences observed in sperm parameters between the two study groups post-intervention. These results may be due to the differences in levels of baseline parameters between the two groups. In their study, treatment group had lower baseline percentage of motile spermatozoa, progressive motile spermatozoa, and the percentage of spermatozoa with normal morphology in comparison to the placebo group. Moreover, Amini et al. in their study investigated the effects of supplementation of VD3 50,000 IU tablets once a week for a 8-week period, as well as a maintenance dose of VD3 within 4 weeks (50,000 IU VD3 supplement monthly) on 62 infertile men with the impaired spermatogonial tests. They also showed that the intake of VD3 did not change the quality and quantity of semen parameters [81]. In the present study, despite the changes in 25(OH) VD3 and calcium homeostasis, no statistically significant differences were observed in terms of semen volume, sperm count, and sperm with normal morphology between the two groups. Accordingly, these results are similar to the previous two interventional studies, but these are in contrast with the results of the Deng XL et al.’s study, reporting positive effects of VD3 and calcium on sperm count.

Although all these previous studies had a great dissimilarity in their plans, including study participants and variability in the selection of patients with spermogram abnormalities, methodology, and cut off values of VD3 status, the presence of some confounders, including co-morbidities, age, and medical treatment was shown to affect semen quality and hormonal therapies that may influence the results of the studies. It is also possible that the effect of VD3 supplementation on semen parameters may be more effective under conditions with severe VD3 deficiency than mild VD3 deficiency or insufficiency. Because based on previous studies conducted on both men and animals, impaired male fertility was found to be linked with severe VD3 deficiency [9, 18, 28, 73].

In the present study, a comparison among the means of hormonal tests such as serum OCN, LH, FSH, T, E2, SHBG, PRO, FAI, T/E2, and T/LH ratios, in those men who participated in the supplement group, demonstrated a statistically significant difference in the T/LH ratio compared to the baseline and to the placebo group.

Stimulation of T production in Leyding cells can be mediated by LH via the increased CAMP production and intracellular calcium ion (Ca^2 +^) ions. There is an evidence suggesting synergistic effects of LH and VD3 on the synthesis of T. VD3, which can also be effective on modulating this process via modulating the calcium-dependent LH response [82]. The ratio of T to LH is known as the best indicator of both LH sensitivity and function of Leyding cells. Mice in which VD3 receptors were removed, showed the reduced T to LH ratios and they also had Leyding cells (resulting in a low T to LH ratio) hyperplasia [71]. In the present study, although VD3 intake indicated no significant effect on testosterone levels, it increased T/LH ratio. This result could confirm the proposed mechanism for the calcium mediated effects of VD3 on the increased sensitivity of Leyding cells to LH and T productions.

Data from the previous studies on the consequences of VD3 on serum levels of sex hormones, especially T and SHBG levels, are controversial and under debate yet. These controversies may be due to the different study methodologies; difference in size, age, BMI, diseases, and levels of 25(OH) VD3; and other confounders, including the time of blood sampling, serum levels of LH, and calcium compared to previous studies.

Based on the previously performed animal studies, VD3 has an indirect effect on T synthesis via genomic VD3-induced expression of osteocalcin (OCN), which is a hormone synthesized by osteoblasts and it is involved in the bone metabolism. It has been shown that in male mice with OCN-deficiency, despite high serum LH amounts, fertility rate, testis size, and specially T levels decreased [83]. The positive correlations between OCN and total T have been also reported in observational studies [84–86]. However, in our study, we observed no positive efficacy of VD3 on increasing OCN levels that may be known as one of the possible reasons for the lack of change in testosterone levels. To the best of our knowledge, this work is the first clinical study that investigated the effects of VD3 supplementation on serum OCN levels among infertile men. Therefore, more large randomized clinical trials are needed in this area.

The age, BMI, and comorbidities were found as the important confounding factor affecting the results of studies performed on the relationship among VD3, SHBG, and T levels. The positive relationship among serum 25(OH)VD3, T, and FAI have been specially observed in men aged over 40 years old, with an average BMI above 25 as well as the presence of metabolic diseases, including metabolic syndrome, diabetes or cardiovascular disease [29, 30, 87, 88]. In contrast, serum 25(OH)VD3 levels were found to be either unrelated or negatively correlated with T and FAI, whereas they were positively correlated with SHBG levels among younger healthy men (18–21 y) [26, 84, 89]. Several studies have previously shown that both serum 25(OH) VD3 and T levels decline with aging, while both gonadotropins and SHBG increase. The age-related alterations may be of clinical importance, which probably are partly responsible for independent influence on VD3 assessment and circulating T levels. So, up to date, inadequate data are available to establish a certain relationship between VD3 status and T levels. Moreover, the association between the increased 25(OH) VD3 and the elevations of total T and SHBG, reduced after the adjustment for BMI in some studies [87, 89]. Of note, the increased BMI is an indicator of obesity [90]. Obesity is associated with the decreased T level, mediated by the reduced SHBG level due to insulin resistance [91]. Lower T also causes obesity [91, 92], and one study revealed that a higher BMI caused the decreased 25(OH) VD3 level; however, a lower 25(OH)VD3 did not cause the increased BMI [93]. These observations suggest a complex role of BMI in the relationship between T and VD3.

The results of the present study are in line with more interventional studies, showing no positive effects of VD3 supplementation on the levels of T. A very short-duration (4 days) and short-duration (12 weeks) in studies performed on the supplementations with VD3 had no significant effects on serum total T levels [94, 95]. Two others long (12 months supplementation) [96] and very long duration of studies (24 months supplementation) did not find any relationship [97]. While, the long supplementation (12 months) with VD2 and VD3 in different-age men groups significantly increased serum total T [98], free T, and SHBG [99]. The very short- term duration of some studies, low dose of VD3 supplementation, and different characteristics of studies’ participants may be the probable reasons responsible for these controversial results. Furthermore, as mentioned earlier and also based on a recent longitudinal study, weight changes are known as one of the main determinant factors in the regulation of T levels [100]. In our study, we also observed no significant changes in BW, BMI, BF%, and WC during the study period that were consistent with the results of a small clinical study investigating the efficacy of 12 months supplementation with 3332 IU cholecalciferol along with weight loss program on SHBG and T levels in 54 obese men with average age of 48 years old [99]. As a result, they reported no changes in BW, while an increase was observed in SHBG and T levels that may be due to the long term duration of the study. Therefore, the link between VD3 and serum androgen levels still remains obscure and controversial, and more investigations are needed to elucidate the relationship between serum VD3 and serum testosterone concentrations.

Studies’ outcomes regarding the influences of VD3 on serum E2 levels are contradictory. No significant relationship was observed between serum E2 and 25(OH) VD3 levels in some cross-sectional studies conducted on healthy young men [22, 26, 84]. While, negative correlation was seen among serum E2, 25(OH) VD3, and ionized calcium in various research [87, 101, 102]. There is an evidence suggesting that VD3 is one of the most important regulators of aromatase gene expression “T-to-E2 converter” and plays an important role in spermatogenesis. Correspondingly, it has been shown that this effect is tissue-specific manner because of the different activities of aromatase promoters (1.3, 1.4, II) in various tissues. Accordingly, this enables VD3 to stimulate the function of aromatase in bone and gonad tissue, while it represses its function in both adipose and breast tissues [101]. Serum E2 greater than 80% in men is mainly originated from adipose tissue conversion of circulating T [102]. So, excess body fat may consequently increase serum estradiol levels [103]. As VD3R suppresses aromatase function in adipose tissue, which may thus decrease bioavailable E2 [102]. In this regard, VD3 supplementation has been suggested as a potential therapeutic option in those programs related to the weight loss in a recent systematic review and meta-analysis of randomized controlled trials [104]. Therefore, this may affect serum E2 levels by decreasing body fat. However, in the present study, no significant changes were observed in mean levels of E2, T/E2 ratio, and BF%. These results may be due to the small sample size, short term duration or low doses of VD3 supplementation, because the reduction in serum E2 levels and BF% and an increase in T/E2 ratio were all nonsignificant. Recently, it has been shown that the increased T/E2 ratio is more important for male fertility than T or E2 levels alone [105]. In the Andersson et al.’s study, low T/E ratio was observed in great population of infertile men. The increased aromatase activity due to hyperstimulation by the increased LH levels was mentioned as the probable underlying mechanism in this regard [106]. However, there is a need for performing more large randomized clinical trials, in order to prove VD3 supplementation effects on spermatogenesis and sex hormones regulation.

Strengths of the present study are the design of the study, the daily dose of VD3 that was effective on raising serum levels of 25(OH)VD3 to adequate levels, the exclusion of the patients who were smoking or had a specific disease, and the control of some confounding factors, including BW, BMI, BF%, WC, physical activity, season, dietary calorie, calcium, F, VD3, selenium, vitamin C, E, A, zinc, serum 25(OH)VD3 and PTH, and baseline values for variables. It should be noted that sampling initiated during the fall, and most of the samples were obtained during this season. Moreover, the intervention was conducted during the winter, which was applied to both groups.

However, further studies with larger sample sizes and longer duration are needed to confirm the clinical efficacy of VD3 supplementation on male infertility. Furthermore, failure to evaluate fertility rate successfully and impossibility to include healthy individuals in the study were the limitations of the present study. In addition, strict inclusion and exclusion criteria such as the absence of any other disease limited generalizability of the results of this study to most cohorts of infertile men.

## Conclusion

In this study, a 3-month supplementation with 4000 IU/day VD3 significantly increased serum 25(OH) VD3, PTH, phosphorus, and seminal and serum calcium; T/LH ratio; and total and progressive sperm motilities in infertile men with asthenozoospermia. However, in the present study, we found no significant effects of VD3 supplementation on reproductive hormones. Altogether, according to the high prevalence of VD3 deficiency worldwide and available evidence on VDR-mediated positive action of serum VD3 on the testis, as well as on spermatozoa, our present data showed that the increased serum 25(OH)VD3 concentration could improve total and progressive sperm motilities, as one of the most predictive value for natural conception and even intrauterine insemination (IUI). This could also improve serum and seminal calcium levels that are important for sperm motility. In addition, it is suggested that VD3 supplementation as a simple, less invasive, and cost effective clinical measurement, with strong biological actions, could be considered as part of a therapeutic strategy designed to improve male fertility. However, further investigations, including randomized, controlled, and double-blinded clinical trials as well as in vitro and in vivo studies, are needed to explore the therapeutic potential of VD 3 supplementation fully in cases of male infertility.

## Data Availability

Datasets are available through the corresponding author upon reasonable request**.**

## Abbreviations

ANCOVA: Analysis of covariance
BMI: Body mass index
BW: body weight
BF: body fat
WC: waist circumference
DNA: Deoxyribonucleic acid
E2: Estradiol
ELISA: Enzyme-linked immunosorbent assay
FSH: Follicle-stimulating hormone
IPAQ: International physical activity questionnaire
LH: Luteinizing hormone
MDA: malondialdehyde
OS: oxidative stress
PTH: parathyroid hormone
RCT: Randomized controlled clinical trial
SHBG: sex hormone-binding globulin
25(OH)VD3: 25 hydroxy vitamin D3
WHO: World Health Organization
